# Population Genetics of an Endemic Species *Mongoloniscus sinensis* (Isopoda: Oniscidea) and Diversification Dynamics Across Northern China

**DOI:** 10.1002/ece3.72318

**Published:** 2025-10-11

**Authors:** Xue Dong, Jianzhi Wu, Xin Zhang, Yao Zhang, Shu Ma, Lingxiu Liang, Wangchuan Zhang, Xia Lu, Jianmei An

**Affiliations:** ^1^ Shanxi Province Engineering Research Center of Protection and Utilization of Endemic Animal Resources School of Life Science, Shanxi Normal University Taiyuan China

**Keywords:** demographic history, gene flow, genetic diversity, mitochondrial genes, phylogeographic break

## Abstract

Endemic to temperate northern China, *Mongoloniscus sinensis* is a soil‐dwelling isopod that provides a powerful system for investigating how Pleistocene climatic fluctuations and mountainous barriers have shaped genetic divergence and regional endemism. Through fine‐scale phylogeographic and demographic analyses, we explored the evolutionary processes shaping genetic differentiation and biodiversity in this region. We obtained three mitochondrial genes (COI, ND5, and 12S rRNA) in 305 specimens collected from 48 localities spanning the species' distribution. Population structure analyses identified 3 clusters corresponding to geographic locations—East, West, and Midland. The Midland group exhibited the highest genetic diversity, possibly representing ancestral populations, followed by the West group and the lowest in the East group. Limited gene flow was observed between eastern and western populations, likely because of the geographical barrier formed by the Taihang and Luliang Mountains, which restrict genetic exchange. Demographic history analyses showed recent expansion in both East and West groups. Divergence time estimation dated the following splits from the Midland group: West (~0.63 Ma) and East (~0.53 Ma), coinciding with Pleistocene climatic oscillations. Ecological niche modeling revealed that *M. sinensis* currently occupies a wide climate range with highly suitable areas in northern China, and its distribution during the LGM was affected by the ice age. For the climate variables, the mean temperature of the coldest quarter was the most important variable to determine species distribution, followed by precipitation of the coldest quarter and elevation, indicating that cold adaptability may constrain the species to northern China. As an important geographical barrier, the Qinling Mountains have a diverse climate and habitats, which play a role in limiting the distribution of species.

## Graphical abstract

We explored the population genetics and demographic history of an isopod species endemic in temperate China on the basis of three mitochondrial genes. Three clusters were identified. The Midland group has the highest genetic diversity, and the greatest differentiation between the East and West groups was due to the geographical barrier caused by the Taihang Mountains and Luliang Mountains. The divergence of the West and East group from the Midland group was influenced by Pleistocene climatic oscillations.

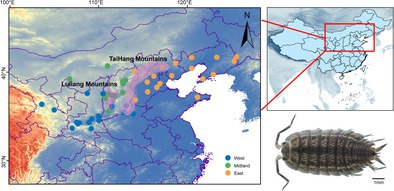

## 1 Introduction

Investigating the mechanisms shaping the present patterns of species distribution is a central goal in biogeography and evolutionary biology (Wiens and Donoghue 2004). The evolutionary trajectories and genetic differentiation of extant species have been profoundly influenced by historical geographic isolation, often driven by mountain uplift and climatic fluctuations during the Pleistocene glacial cycles (Hewitt 2000, 2004). In particular, repeated glacial–interglacial cycles since the Pleistocene Epoch induced large‐scale range contraction/expansion in numerous plants and animal taxa (Clark and Mix 2002; Hewitt 2000). These range dynamics, often coupled with population bottlenecks and genetic drift, fostered genetic differences and led to the formation of distinct phylogeographic patterns (Petit et al. 2003; Webb and Bartlein 1992). Mitochondrial DNA (mtDNA) has been widely employed in phylogeography and population genetic studies because of its maternal inheritance, much faster mutation rate compared to nuclear genomic sequence, and a lack of genetic recombination (Allio et al. 2017; Remi et al. 2017). These properties make mtDNA particularly useful for reconstructing evolutionary histories and inferring historical demography (e.g., Haag‐Liautard et al. 2008; Muraro et al. 2024; Pakendorf and Stoneking 2005; Promnun et al. 2021; Rato et al. 2024).

The suborder Oniscidea (commonly known as woodlice) is the only group within Crustacea that is almost entirely composed of terrestrial species and represents the most diverse and abundant taxa within Isopoda (Schmidt 2008; Sfenthourakis and Taiti 2015). Although isopods are globally distributed (Paoletti and Hassall 1999), they exhibit limited dispersal capacity because of their low vagility and dependence on moist microhabitats (Taiti 2004), making them a fascinating model for investigating fine‐scale phylogeographic patterns and historical diversification processes (Karagkouni et al. 2016). Recent phylogeographic studies on isopods have predominantly focused on island and aquatic species (Vieira et al. 2019). To date, only one study has examined the phylogeographic patterns of a terrestrial isopod ( *Armadillo officinalis* ) in the Mediterranean region (Dimitriou et al. 2022), revealing how paleogeographic history and recent human activities influenced its genetic structure.

**FIGURE 1:**
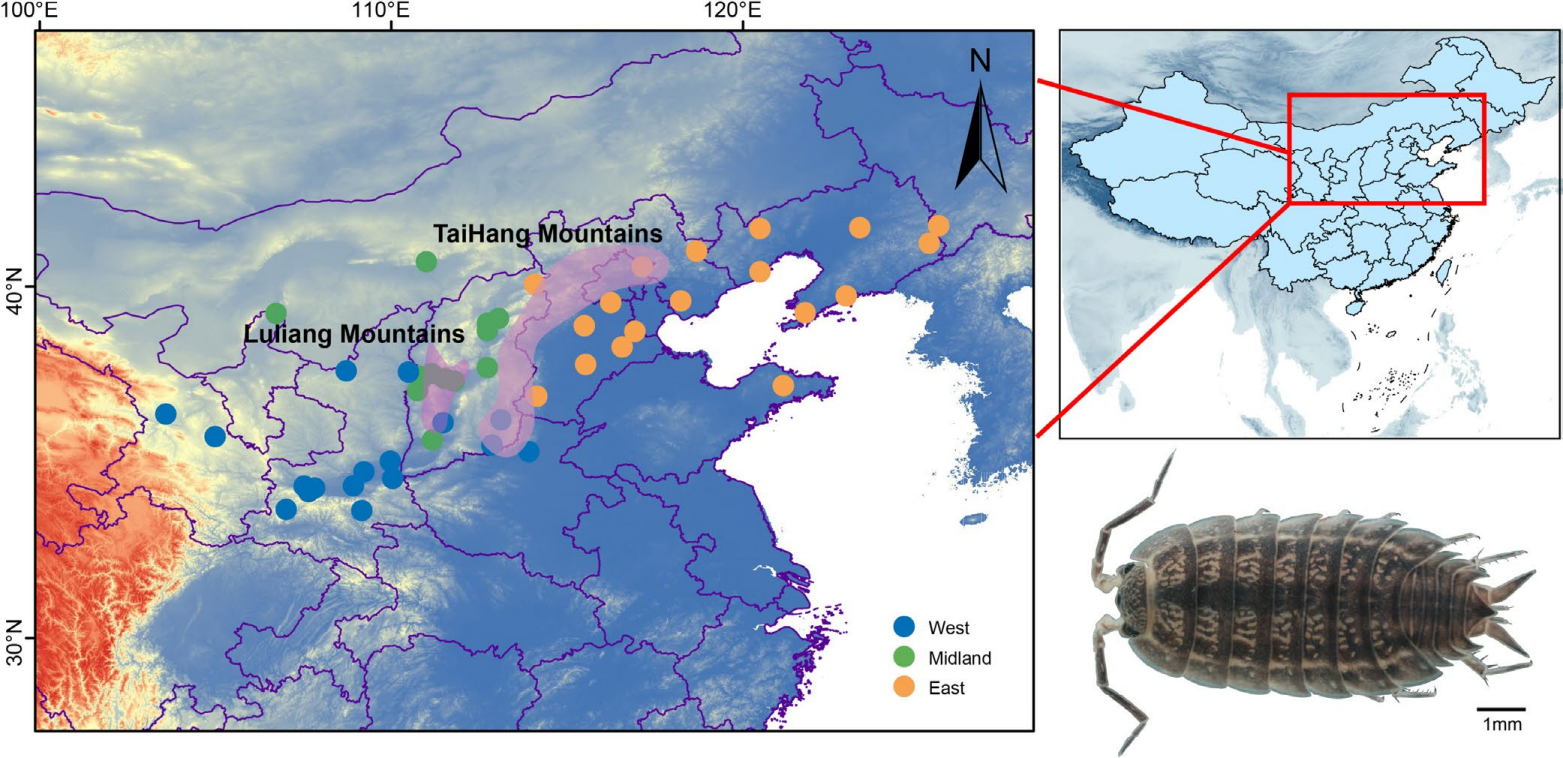
Map depicting the geographic location of the sampled populations of *M. sinensis* . The sample locations in Table 1 are colored according to the genetic lineages, which are color‐coded by population structure in Figure 2.

Here, we focus on *Mongoloniscus sinensis* (Dollfus, 1901) (Crustacea: Isopoda: Oniscidea: Agnaridae), a soil‐dwelling isopod endemic to the temperate regions of northern China (Figure 1) (Chen 2000). *M. sinensis* plays an important ecological role because of its ability to tolerate and accumulate high levels of heavy metals such as Zn and Cd (Zhao et al. 2019), making it a valuable bioindicator of soil contamination. A preliminary population genetic study on the basis of mitochondrial COI sequences revealed evidence of genetic diversity in *M. sinensis* (Zhao et al. 2016), but the study was limited in its geographic scope and did not assess broader phylogeographic patterns or historical processes. A comprehensive phylogeographic investigation with extensive spatial sampling is therefore essential to clarify its evolutionary history and to contribute to a broader understanding of biogeographic diversification within Oniscidea and Agnaridae.

In contrast to extensive phylogeographic studies from the Qinghai‐Tibet Plateau and subtropical China (Qu et al. 2010; Tang et al. 2022; Ye et al. 2020), relatively few taxa have been addressed in northern China. Only limited studies have explored biogeographic patterns in this region, including work on plants (Xu et al. 2019; Lin et al. 2021; Zong et al. 2017), spiders (e.g., *Sinothela* and *Ganthela*, Xu et al. 2018), and lizards (e.g., *Eremias argus* and *E. brenchleyi*, Zhao et al. 2011). This highlights a gap in our understanding of biogeographic diversification across taxa in northern China. Several prominent mountain ranges in northern China—such as the Taishan, Taihang and Luliang Mountains—have been proposed as historical phylogeographic barriers that restricted gene flow and contributed to lineage divergence (Ma et al. 2024; Xu et al. 2018; Zhao et al. 2011). Notably, the Taihang Mts (extending from 35° to 40° N) form a natural demarcation line between the second (altitude 1000–2000 m) and third (altitude < 500 m) steps of Chinese topography. It has been identified as a potential phylogeographic boundary and biodiversity center because of its ecological and climatic significance (Fu et al. 2018; Geng et al. 2018; Zhao et al. 2017; Zhu et al. 2008). The region is characterized by highly heterogeneous topography, soil types, climate, and vegetation and is home to a high level of species richness and may have provided glacial refugia during the Quaternary (Ma et al. 2024). Similarly, the Luliang Mts—stretching north–south across western Shanxi—are thought to have shaped local endemism biotas (Hou et al. 2014).

In this study, we investigated the phylogeography of *M. sinensis* , a terrestrial isopod endemic to temperate northern China. We sampled 305 individuals from 48 localities, covering most of the species' known distribution. Phylogenetic and population genetic analyses were performed using three mitochondrial markers (COI, ND5, and 12S rRNA), in combination with ecological niche modeling. Our primary goals were to (1) characterize the phylogeographic pattern of *M. sinensis* across its range and (2) evaluate the extent to which paleogeographic events and Quaternary climatic fluctuations have shaped the genetic diversity and population structure.

## 2 Materials and Methods

### 2.1 Sampling and Laboratory Procedures

We collected 305 individuals from 48 sampling sites across the native range of *Mongoloniscus sinensis* in northern China. The geographic location of each locality is represented in Figure 1, and detailed information is in Table 1. All specimens were preserved in 100% ethanol and stored at −20°C in the School of Life Science at Shanxi Normal University (Shanxi, China). Genomic DNA was extracted from the pereopods using the Animal Tissues/Cells Genomic DNA Extraction Kit (Solarbio, China). Three mitochondrial markers were amplified for all individuals: 12S small subunit rRNA (12S), cytochrome c oxidase subunit I (COI), and NADH dehydrogenase subunit 5 (ND5). PCR primers were adopted from previously published studies (Podsiadlowski and Bartolomaeus 2005; Ren et al. 2005), and the PCR amplification procedures are listed in Table S1. Sequencing was conducted in both directions by Sangon Biotech (Beijing, China) using Sanger sequencing technology. Chromatograms were imported into Geneious 2020.2.1 (Kearse et al. 2012; available at http://www.geneious.com) for manual inspection. Putative heterozygous sites were assessed on the basis of quality score and verified by visual inspection of the chromatograms. No double peaks were observed, and all sequences were considered authentic mitochondrial haplotypes. Sequences were aligned using the MAFFT 7.402 (Katoh and Standley 2013) with the G‐INS‐I strategy. Poorly aligned regions were removed using GBLOCKS (Talavera and Castresana 2007). The final alignments included COI (603 bp), ND5 (375 bp), and 12S rRNA (456 bp) and were concatenated using PhyloSuite 1.2.2 (Zhang et al. 2019).

**TABLE 1:** Sample collection and haplotype polymorphisms in each geographical population of *Mongoloniscus sinensis* across the distribution areas in China on the basis of concatenated mitochondrial data.

Sampling site	Latitude (°N)	Longitude (°E)	Collection date	Number of samples	S	NHap	Hd
JLTH	41.73	125.57	2019.08	3	0	1	0
LNBX	41.23	125.30	2019.08	2	0	1	0
LNSY	41.67	123.33	2019.08	6	0	1	0
LNCY	41.65	120.49	2019.08	11	1	2	0.327
LNHLD	40.42	120.49	2017.06	8	13	4	0.643
LNSSLB	39.25	121.77	2019.08	3	4	3	1.000
LNZH	39.73	122.94	2019.08	7	8	5	0.905
SDYT	37.18	121.15	2017.06	11	25	8	0.927
TJTGT	38.72	116.93	2019.08	8	7	5	0.786
BJDC	39.54	116.24	2017.06	8	8	2	0.250
BJMY	40.56	117.14	2017.06	5	0	1	0
HEBCD	41.00	118.69	2019.08	11	6	4	0.491
HEBTS	39.59	118.23	2019.08	1	—	—	—
HEBCZ	38.28	116.57	2019.08	6	2	3	0.600
HEBHS	37.78	115.53	2019.08	4	10	3	0.833
HEBBD	38.88	115.49	2018.06	4	2	2	0.667
HEBZJK	40.06	114.08	2018.06	3	2	2	0.667
HEBXT	36.88	114.14	2019.08	5	2	3	0.700
HNXX	35.30	113.91	2017.10	10	19	2	0.200
NMHH	40.70	111.00	2023.07	4	23	4	1.000
SXXG	38.74	112.73	2023.07	3	10	3	1.000
SXXS	38.95	112.73	2023.07	3	69	3	1.000
SXXD	39.09	113.05	2017.07	6	95	3	0.600
SXLZ	37.30	111.79	2023.07	3	36	3	1.000
SXLQ	37.38	111.51	2023.07	3	91	3	1.000
SXLLX	37.45	110.75	2023.07	4	92	4	1.000
SXLL	37.40	110.87	2017.08	3	49	3	1.000
SXLX	37.52	111.14	2017.08	7	85	5	0.857
SXLSL	37.03	110.75	2017.08	12	107	9	0.939
SXJQ	37.69	112.73	2017.10	6	77	4	0.800
SXCZ	36.20	113.12	2017.10	11	19	7	0.818
SXLF	36.12	111.47	2017.06	20	63	13	0.926
SXJC	35.49	112.86	2017.10	9	17	5	0.833
SXYC	35.63	111.17	2017.10	9	95	6	0.917
SHXYS	37.57	110.49	2023.07	4	17	4	1.000
SHXYJ	37.60	108.72	2023.07	2	22	2	1.000
SHXWH	34.54	110.04	2021.05	13	14	10	0.923
SHXWC	35.03	109.97	2021.05	10	7	6	0.889
SHXWF	34.73	109.22	2021.05	11	6	4	0.491
SHXXA	34.33	108.92	2017.10	7	24	6	0.952
SHXBM	34.27	107.82	2021.05	7	3	4	0.714
SHXBB	34.32	107.52	2021.05	2	1	2	1.000
SHXBT	34.16	107.65	2021.05	10	6	5	0.667
SHXHL	33.66	107.01	2021.05	8	16	8	1.000
SHXSL	33.63	109.16	2021.05	4	3	3	0.833
NXSH	39.23	106.72	2023.07	3	15	3	1.000
GSLZ	36.37	103.59	2023.07	2	4	2	1.000
GSBQ	35.73	104.99	2023.07	3	4	3	1.000

*Note:* Hd, haplotype diversity; Nhap, number of haplotypes; S, number of segregating sites.

### 2.2 Genetic Polymorphism, Phylogenetic Analysis, and Population Genetic Structure

Genetic diversity indices for the concatenated mitochondrial data were measured by the number of polymorphic sites (*S*), number of haplotypes (Nhap), haplotype diversity (*Hd*), and nucleotide diversity (*π*), which were calculated in DnaSP v5.0 (Librado and Rozas 2009). A phylogenetic analysis was performed using the Neighbor‐Net (NN) approach in SplitsTree v4.14.5 (Huson and Bryant 2006) that created a distance matrix on the basis of pairwise uncorrected P distances. Bayesian Analysis of Population Structure (BAPS v6.0; Cheng et al. 2013) was used to infer genetic clustering, integrating geographic coordinates and genetic data to estimate the optimal number of genetic groups (K = 1–10). The best K value was selected on the basis of the highest marginal log‐likelihood value from 10 replicates. A haplotype network was constructed for the concatenated dataset using the TCS algorithm in PopArt v1.7 (Leigh and Bryant 2015) to visualize relationships among haplotypes.

Pairwise F_st_ values were calculated, and hierarchical analysis of molecular variance (AMOVA) was conducted using Arlequin 3.5 (Excoffier and Lischer 2010), with K = 3 (from BAPS, see results) and 5000 permutations to quantify genetic variation.

### 2.3 Historical Demographic and Divergence Time Estimation

Historical demography for each population group and all populations of *M. sinensis* were assessed by mismatch distribution analysis (Rogers and Harpending 1992) in DnaSP v5.0 (Librado and Rozas 2009). Tajima's D (Tajima 1989) and Fu's Fs (Fu 1997) were calculated to further estimate potential expansion events. To ensure accurate divergence time estimation, we excluded samples from localities showing mixed haplotype group affinities in the network. Divergence time was estimated in BEAST 2.6.2 (Bouckaert et al. 2014) using a strict clock model and a constant‐size coalescent prior. The time scale was calibrated using the COI substitution rate (1.56%–1.72%/Ma; Brandão and de Mello 2018), previously estimated for Oniscidea. Markov Chain Monte Carlo (MCMC) chains were run for 10 million generations, sampling every 1000 generations. Convergence and effective sample size (ESS > 200) were confirmed using Tracer 1.6 (Bouckaert et al. 2014). The maximum clade credibility tree was determined in TREEANNOTATOR v.2.4.1 (Drummond and Rambaut 2007) after discarding the first 25% of the trees as burn‐in.

### 2.4 Gene Flow Estimation

To infer the rates and direction of gene flow, we used MIGRATE v4.4.3 (Beerli and Felsenstein 2001) to estimate the number of migrants per generation in population. Individual assignments were based on the above genetic structure results. The parameters were set as follows: long‐inc = 100, long‐steps = 1000,000, burn‐in = 100,000. Four independent MCMC runs were conducted for a million generations, and the first 10% samples were discarded as burn‐in. Consistency among the three independent MCMC estimates, along with a smooth and unimodal distribution over a prior range for all three estimations, was viewed as a sign of convergence. The best model was selected by comparing the marginal likelihoods of three models with different gene flow directions.

### 2.5 Species Distribution Model of Present and Past

We used 48 occurrence records from our field work to model the present and past distribution of *M. sinensis* . To reduce the effect of spatial autocorrelation, 43 localities separated from each other by more than 50 km were retained for the species distribution model (SDM). Nineteen bioclimatic variables from the WorldClim database (http://www.worldclim.org/) at a resolution of 2.5‐arc were used, of which seven were retained after removing highly correlated variables (|*r*| > 0.80): mean diurnal temperature range (BIO2), isothermality (BIO3), maximum temperature of the warmest month (BIO5), mean temperature of the coldest month (BIO11), precipitation of the wettest month (BIO13), precipitation seasonality (BIO15), and precipitation of the coldest month (BIO19). The maximum entropy was implemented in Maxent 3.4.4 (Phillips et al. 2006) to predict the species distribution range of *M. sinensis* . The analysis was run using the default settings, with 75% of the records for training and 25% for model testing (Corbalán et al. 2011). The model was projected onto the climatic conditions of the Last Glacial Maximum (LGM) simulated by the Community Climate System Model 3 (CCSM3). Model performance was evaluated using the areas under the curve (AUC) of the receiver operating characteristic (ROC) plot.

## 3 Results

### 3.1 Genetic Polymorphism and Phylogenetic Analyses

**FIGURE 2:**
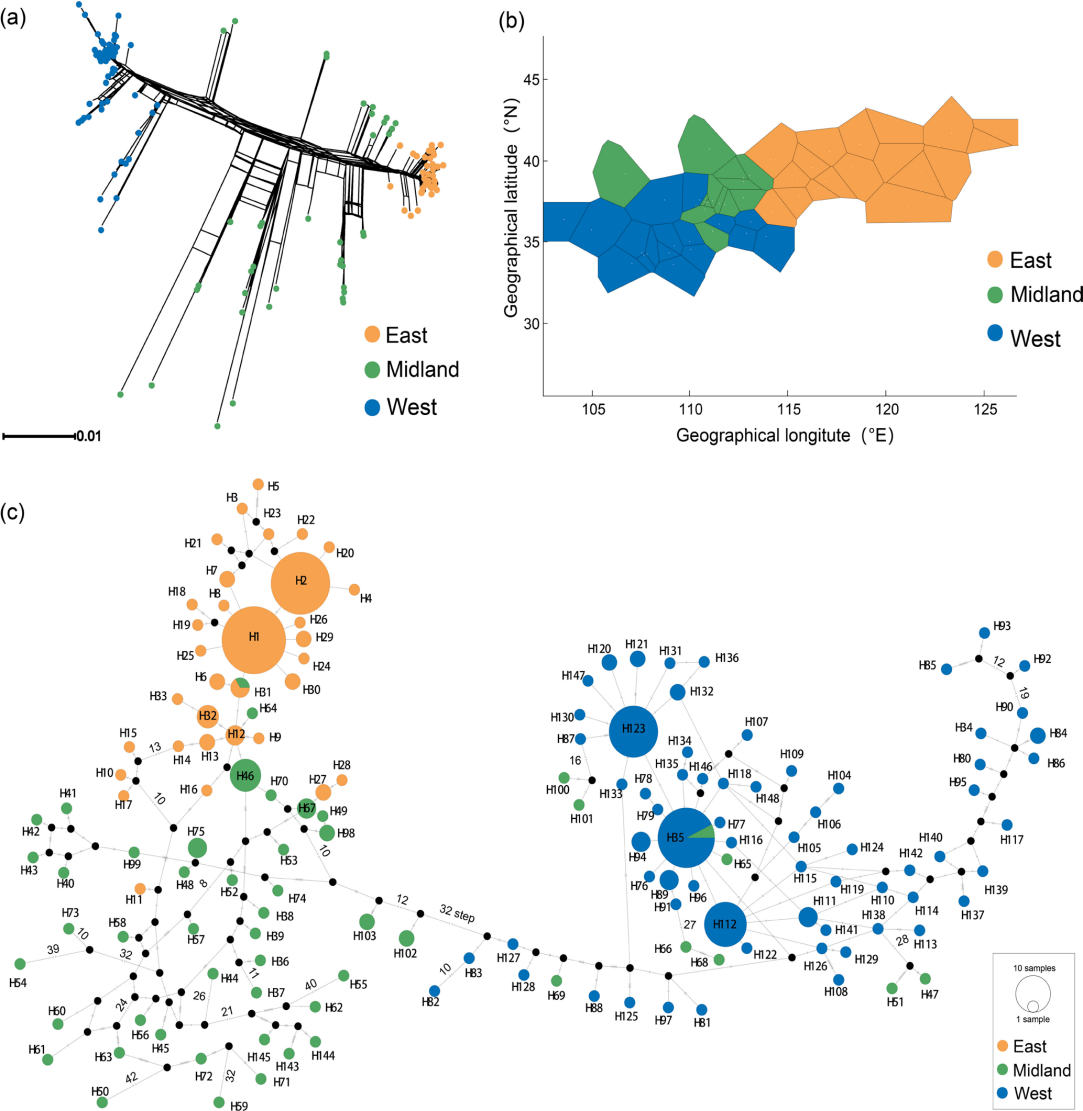
(a) Neighbor‐net network tree constructed in SplitsTree using uncorrected P distance. Colors correspond to the three genetic groups identified by BAPS clustering: East (orange), Midland (green), and West (blue). (b) BAPS clustering result showing the population genetic structure of *M. sinensis* , with K = 3 inferred groups. (c) Haplotype network of *M. sinensis* on the basis of the concatenated mitochondrial genes (COI + ND5 + 12S rRNA). Each circle represents a unique haplotype, and circle size is proportional to the number of individuals sharing that haplotype. Lines represent mutational steps, and black dots indicate hypothetical intermediate haplotypes not sampled.

We generated 915 new mitochondrial sequences from 305 individuals, targeting three gene segments: COI, ND5, and 12S genes. The concatenated sequence totaled 1434 base pairs and comprised 148 mitochondrial haplotypes. The haplotype diversities (Hd) ranged from 0.000 to 1.000 (Table 1). The Neighbor‐Net phylogenetic tree revealed three genetic lineages corresponding to the East, Midland, and West groups, as defined by the BAPS clustering and their geographic locations (Figure 2a). These lineages are color‐coded according to BAPS results (see Section 3.2). Nucleotide polymorphism for the three groups is summarized in Table S2. The Midland group exhibited the highest haplotype and nucleotide diversity (Hd = 0.982; π = 0.0347), despite its smaller sample size than the other groups, whereas the East group showed the lowest level of diversity (Hd = 0.823; π = 0.0029).

### 3.2 Population Genetic Structure

A total of 148 haplotypes were identified from all 305 samples. In the haplotype network (Figure 2b), the East and West groups are far apart from each other, indicating pronounced genetic differentiation. The haplotypes of the Midland group, located centrally in the network, likely represent ancestral haplotypes, whereas East and West haplotypes appear derived. The Midland network was more reticulated, suggesting a longer history or more complex evolutionary process. BAPS identified three major genetic clusters, geographically corresponding to East (e.g., Liaoning, Beijing, Tianjin, Hebei, Shandong), West (e.g., Shaanxi, southern Shanxi, Gansu), and Midland (central/northern Shanxi and Inner Mongolia) populations (Figure 2c). AMOVA revealed that 72.62% of the total genetic variance occurred among groups (*p* < 0.01), whereas 20.10% and 7.28% were found within populations and among populations within the group, respectively (Table 2). Pairwise F_ST_ values ranged from 0.3289 to 0.9110 and were all significant (Table 3). The highest level of genetic differentiation was detected between the East and West groups (0.9110), followed by the West and Midland groups (0.6077), whereas the lowest was observed between the East and Midland groups (0.3289).

**TABLE 2:** AMOVA of three population subgroups on the basis of mitochondrial data for *M. sinensis* .

Source of variation	df	Sum of squares	Variance components	Percentage of variation
Among groups	2	4191.102	21.16342 Va	72.62*
Among populations within group	45	858.543	2.12147 Vb	7.28
Within populations	257	1505.647	5.85855 Vc	20.10
Total	304	6555.292	29.14343	

*Note:* Geographic group: group (East, West, and Midland). *p < 0.01.

**TABLE 3:** Pairwise values of genetic differentiation coefficient (Fst, below diagonal) and significance (p, above diagonal) of three mitochondrial sequences among three groups of *M. sinensis* .

	East	Midland	West
East		0.000	0.000
Midland	0.3289		0.000
West	0.9110	0.6077	

### 3.3 Demographical History

**FIGURE 3:**
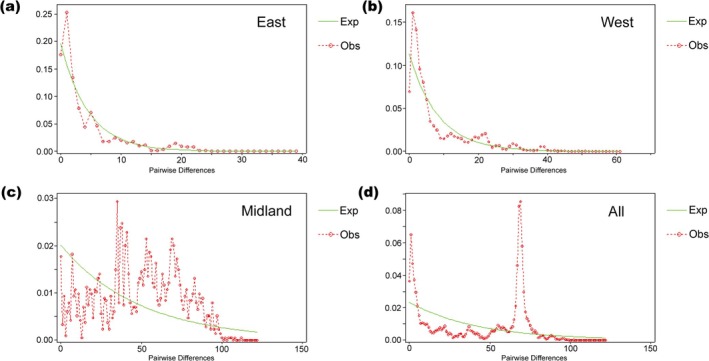
Mismatch distribution of concatenated mitochondrial genes of *M. sinensis* . The line represents distributions expected for expansion and the dotted line represents the observed mismatch distribution. (a) East group; (b) West group; (c) Midland group; (d) all populations.

To assess past demographic dynamics, we performed mismatch distribution analysis and neutrality tests. The observed and expected mismatch distributions for the East and West groups both showed unimodal patterns (Figure 3a,b), indicating the recent demographic expansion. This inference was further supported by significantly negative values of Tajima's D and Fu's F_S_ (Table 4). In contrast, the Midland group and the full dataset showed multimodal distributions in mismatch distribution analyses (Figure 3c,d).

**TABLE 4:** Neutrality tests in defined groups and whole dataset on the basis of mitochondrial DNA data.

	Tajima's D	Fu's Fs
East	−1.9922***	−16.0675***
Midland	−0.2768	−4.4387
West	−1.9474***	−24.6592***
All	−1.4055	−15.0551*

### 3.4 Divergence Time

**FIGURE 4:**
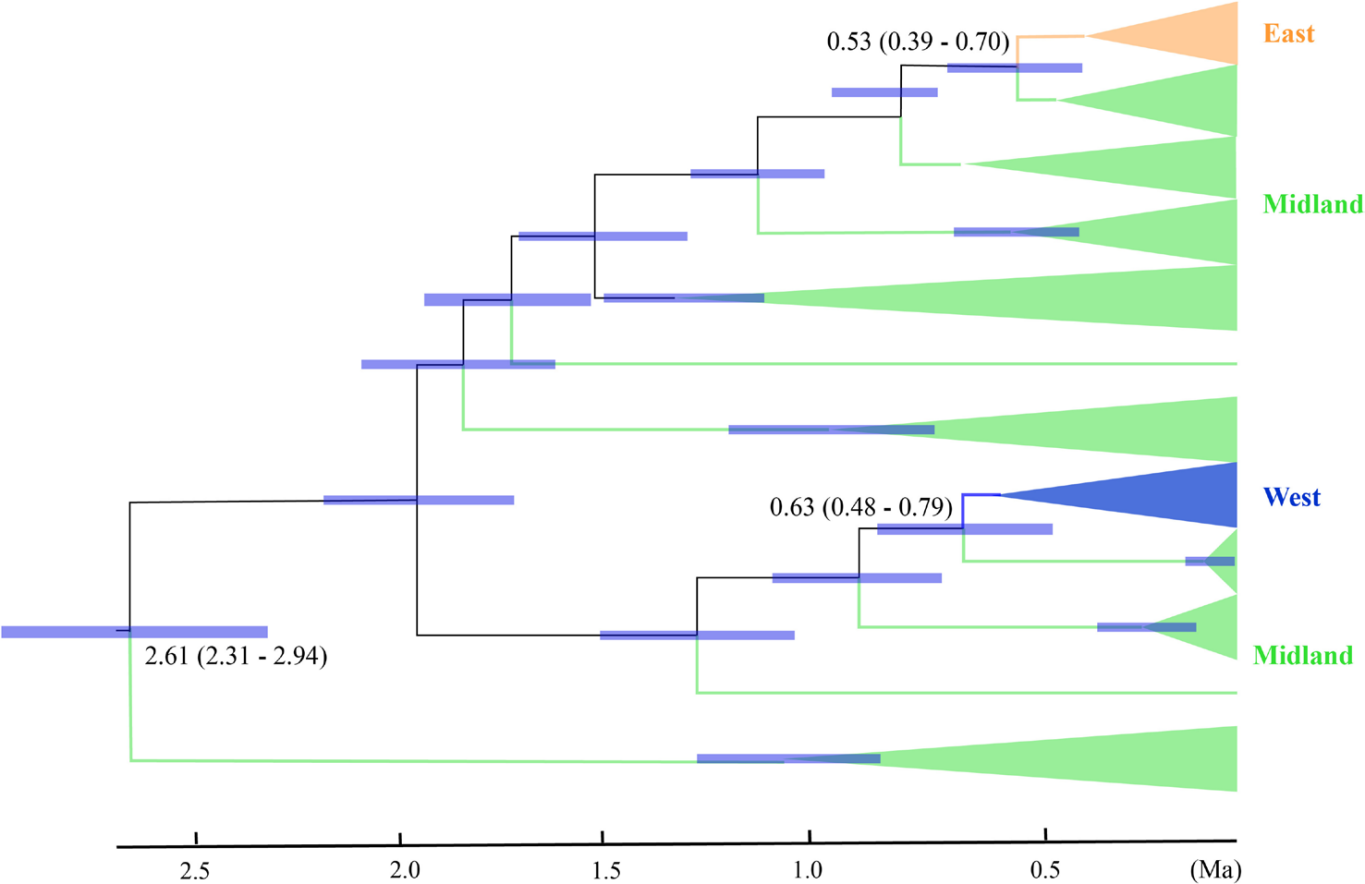
Chronogram of *M. sinensis* derived from the maximum clade credibility tree estimated and implemented in *BEAST. The blue bars indicate 95% highest posterior density (HPD) intervals of the age estimates. Three nodes are highlighted: the node where *M. sinensis* began to diversify and the nodes where eastern and western populations diverged.

Divergence time estimation on the basis of concatenated mitochondrial sequence indicated that *M. sinensis* began to diversify during the late Pilocene, approximately 2.61 million years ago (Ma) (95% HPD: 2.31–2.94 Ma; Figure 4). The East and West groups diverged from the Midland group in the middle Pleistocene, around 0.63 Ma (95% HPD: 0.48–0.79 Ma; Figure 4) and 0.53 Ma (95% HPD: 0.39–0.70 Ma; Figure 4) respectively.

### 3.5 Analysis of Migration Rates

Migrate analysis revealed asymmetric historical gene flow between the Midland and East/West groups. No gene exchange was detected between the East and West groups. The number of migrants was higher from the East to the Midland than in the opposite direction (257.7 vs. 13.0, Table S3), and a similar pattern from the West to the Midland group (68.3 vs. 35.7, Table S3).

### 3.6 Ecological Niche Modeling

**FIGURE 5:**
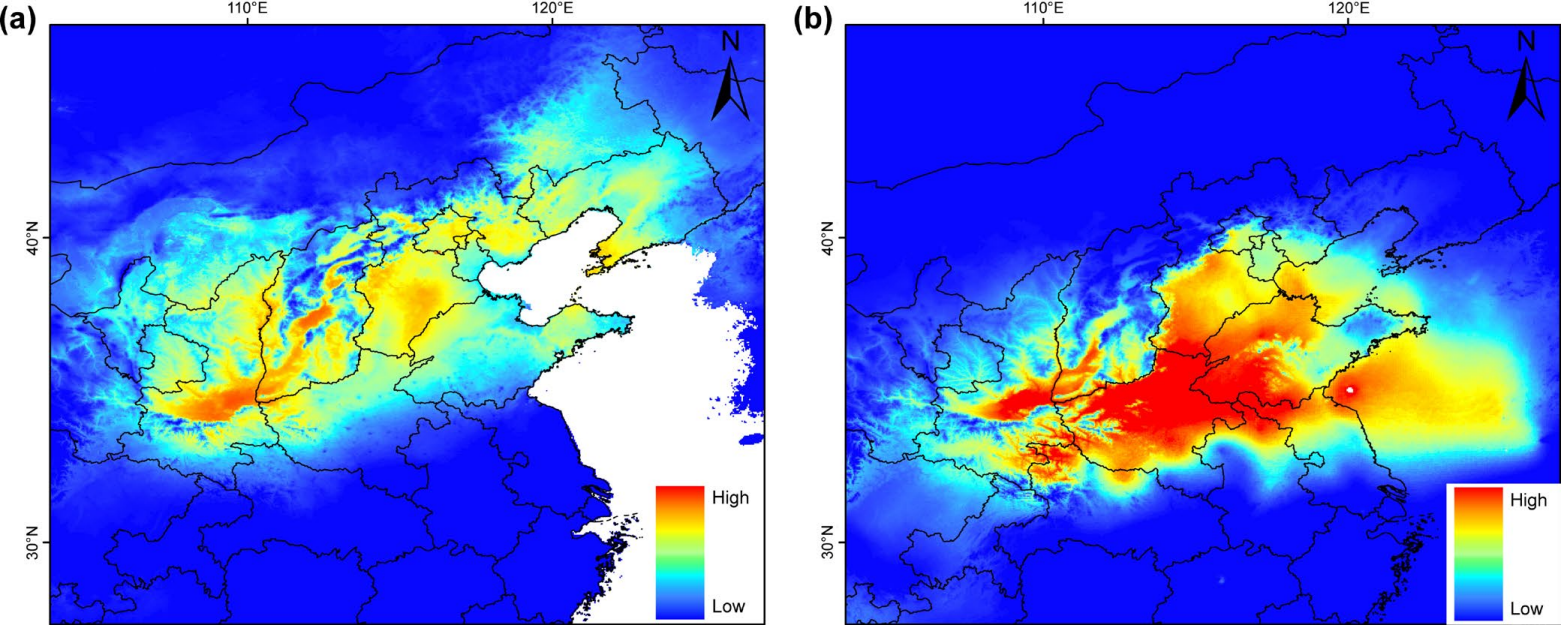
Results of species distribution model in *M. sinensis* . The highest probability of distribution was shown in red color, whereas the lowest probability was shown in blue color. (a) The current suitable areas for *M. sinensis* ; (b) the suitable areas for *M. sinensis* during the LGM.

Ecological niche models exhibited good predictive power, with all models performing well (AUC > 0.8). The predicted current distribution closely matched the observed occurrences, showing a broadly continuous range across northern China (Figure 5a). Under LGM climate conditions, the highly suitable habitats for *M. sinensis* were concentrated in the Southeast of Taihang Mountains (Figure 5b). Northward expansion of suitable habitat from the late Pleistocene to the present suggests that the distribution of *M. sinensis* has been strongly influenced by the glacial cycles. Our results suggest that the species' habitat has remained largely continuous across the temperate zone of China from the LGM to the present (Figure 5).

## 4 Discussion

### 4.1 Genetic Diversity and Population Structure of *M. sinensis*

Phylogeographic and population genetics analyses offer critical insights into the evolutionary processes that shape species distributions and provide an important framework for biodiversity conservation (Cutter 2013). Endemic species, in particular, contribute significantly to regional biodiversity and are often the focus of conservation efforts. In this study, we investigated the genetic structure of *M. sinensis* , an isopod species endemic to the temperate zone of northern China, in order to reconstruct its phylogeographic history. At the population level, the northern populations exhibited relatively higher genetic diversity (Table 1), consistent with previous findings on the basis of COI sequence (Zhao et al. 2016). In the present study, we identified a well‐resolved population structure for *M. sinensis* , comprising three major genetic clusters—East, West, and Midland clades—supported by congruent results from neighbor‐net tree, BAPS analysis, and haplotype network analyses. These analyses consistently demonstrated a clear division between the West and East groups, with the Midland group exhibiting a close relationship with both (Figure 2). Notably, the Midland group showed high haplotype and nucleotide diversity, suggesting the retention of ancestral polymorphisms. The observed patterns were further corroborated by the AMOVA results, which indicated significant molecular variance among groups (Table 2). Pairwise *F*_ST_ values also reflected strong population differences, with the greatest genetic differentiation between East and West groups and relatively lower divergence between the Midland group and the other two (Curnow and Wright 1978) (Table 3), supporting the hypothesis that both East and West populations may have derived from an ancestral Midland population, likely located around the Taihang Mts and Luliang Mts. Gene flow analysis revealed limited connectivity between East and West groups, reinforcing the view that these populations are effectively isolated. Taken together, the observed population structure was likely shaped by a combination of historical isolation because of mountain barriers and low dispersal ability of terrestrial isopods.

### 4.2 Phylogeographical Pattern and Inference of Demographic History

Mitochondrial data strongly supported the existence of three major groups in *M. sinensis* . Demographic reconstructions suggested recent population expansion in both the East and West populations, as evidenced by unimodal mismatch distribution and significantly negative values in the neutral test. In contrast, the Midland group appeared to have maintained a relatively constant population size (Figure 3, Table 4). Two sampling sites in Inner Mongolia (NMHH) and northern Ningxia (NXSH) clustered with the Midland group, although they are far from its core distribution. This pattern may be attributable to anthropogenic dispersal, as field observations indicate that *M. sinensis* is frequently associated with human activities, suggesting a potential for human‐mediated range expansion.

To elucidate the historical processes underpinning the observed phylogeographic structure, we estimated divergence times among major clades. The splits between the Midland clade and the West/East clade were dated to approximately 0.63/0.53 Ma respectively (95% credibility interval: 0.48–0.79 Ma/0.39–0.70 Ma), placing their divergence firmly within the Pleistocene (2.5 Ma–11.7 Ka; Figure 4). This period was marked by repeated glacial–interglacial cycles, which caused frequent climatic fluctuation of the Quaternary Period (G. Hewitt 2000), which likely influenced habitat connectivity, thereby shaping population fragmentation and expansion (de Oliveira et al. 2021; Hawlitschek et al. 2012; Yu et al. 2014). Specifically, although northern China was not covered by ice sheets during the Quaternary Period, it experienced drastic climatic fluctuations (G. Hewitt 2000; Ye et al. 2024). Suitable habitats during the glacial period may have served as refugia, facilitating subsequent post‐glaciation recolonization (Stewart et al. 2010; Chen and Lou 2019). Moreover, the East clade appears to have diverged recently, which may explain its comparatively lower genetic diversity. Geological events and the emergence of physical barriers, such as mountains or rivers, have played a critical role in promoting vicariant divergence by limiting dispersal and gene flow among populations (Hoorn et al. 2010; Xu et al. 2018). In the case of *M. sinensis* , two major phylogeographic boundaries were identified. The first, separating the Midland and the East clades, corresponds to the north‐to‐south extent of the Taihang Mts and is consistent with a known tectonic boundary. Intermittent activity of the QTP, from approximately 3.5 to 1.6 Ma (late Miocene to Pleistocene periods), drove the rapid uplift of Taihang Mts during the Pleistocene (Wu et al. 1999; Lei et al. 2019). With peaks reaching ~2800 m in elevation, the Taihang Mts were characterized by deep gullies and immense valleys (Zhang et al. 2003). This complex topography serves as a significant geographical barrier in northern China, leading to population fragmentation, reduced gene flow, and increased habitat heterogeneity (Liu et al. 2018). The genetic discontinuity observed in *M. sinensis* likely reflects the isolating effect of this mountain (Table 3; Zhao et al. 2013; Chou et al. 2011). The second boundary, separating the Midland and West clades, corresponds spatially to the Luliang Mts, located in western Shanxi Province. This range extends about 350 km and reaches elevations between 1500 and 2830 m. The Luliang Mts have undergone multiple phases of uplift since the Late Cretaceous (Zhao et al. 2009), with their modern topographic configuration forming during the mid–Early Pleistocene (1.8–1.4 Ma) (Li et al. 2013). Similar to the Taihang Mountains, the Luliang range likely contributed to population isolation and lineage divergence through its role as a physical dispersal barrier. Together, these findings support a scenario in which geographic barriers (Taihang and Luliang Mts) and Quaternary climatic oscillations jointly contributed to lineage isolation and regional divergence in *M. sinensis* .

The phylogeographic patterns observed in *M. sinensis* align with broader biogeographic processes recognized across northern China. For example, east–west phylogeographic splits have been identified in species such as the pear *Pyrus betulaefolia* (Zong et al. 2017), the lizards *Eremias argus* and *E. brenchleyi* (Zhao et al. 2011) and the ants *Camponotus japonicus* (Ma et al. 2024). Similar biogeographic patterns shaped by geological features and Pleistocene climatic oscillations have also been reported in other regions and taxa. For instance, lineage diversification in the widespread plant *Elymus sibiricus* across Eurasia has been linked to the uplift of the Qinghai‐Tibetan Plateau and the Pleistocene climate oscillations (Xiong et al. 2024). Likewise, in the Mediterranean region, the pond turtle *Mauremys leprosa* exhibits phylogeographic structure shaped by geographical features such as the Moroccan mountain ranges and the Strait of Gibraltar, in combination with Pleistocene climatic oscillations (Veríssimo et al. 2016).

Although our current study relied solely on mitochondrial markers, the results nonetheless revealed a well‐defined genetic structure across populations. However, mitochondrial DNA has inherent limitations in phylogeographic inference. As a non‐recombining, maternally inherited marker, mtDNA reflects only a single genealogical history, which limits its ability to detect hybridization events and may fail to capture the full picture of population structure shaped by biparental inheritance (Dallaire et al. 2025; Xu et al. 2024). Additionally, the presence of nuclear mitochondrial pseudogenes (NUMTs) may confound analyses if inadvertently amplified (Bensasson et al. 2001). To minimize this risk, we carefully inspected chromatograms to avoid including heteroplasmic or pseudogene‐like sequences. Although our mitochondrial‐based dataset has yielded robust phylogeographic patterns, future work incorporating genome‐wide nuclear SNP data will enhance resolution and explore potential local adaptation mechanisms of *M. sinensis* .

### 4.3 Implications of Endemic Distribution for *M. sinensis*

As a species endemic to northern China, the observed distribution pattern and genetic differentiation of *M. sinensis* are likely shaped by the combined effects of geological history, quaternary climatic fluctuations, and intrinsic biological characteristics. *M. sinensis* is confined to the Palaearctic region, with its southern distributional boundary limited by the Qinling Mountains and Huaihe River (Gao et al. 2017; He et al. 2017; Kreft and Jetz 2013). The Qinling Mountains serve as a prominent topographic and ecological barrier, dividing China into temperate northern and subtropical southern regions. Ecological niche modeling identified the mean temperature of the coldest quarter (bio 11) as the most important variable to determine species distribution (44.5%), followed by precipitation of the coldest quarter (bio19, 20%) and elevation (13.5%) (Table S4), suggesting a strong cold‐adapted attribute of this species. Consistent with our findings, the Qinling Mts have also been recognized as a key phylogeographic barrier across taxa (e.g., Fang et al. 2015; Ma et al. 2024), reinforcing their role in promoting regional endemism by limiting species dispersal.

## Author Contributions

**Jianmei An:** conceptualization (equal), investigation (equal), project administration (equal), resources (equal), supervision (equal), validation (equal), writing – review and editing (equal). **Xue Dong:** conceptualization (equal), formal analysis (equal), investigation (equal), methodology (equal), software (equal), writing – original draft (equal), writing – review and editing (equal). **Jianzhi Wu:** data curation (equal), formal analysis (equal), software (equal), visualization (equal), writing – original draft (equal). **Xin Zhang:** data curation (equal), methodology (equal). **Yao Zhang:** formal analysis (equal), software (equal). **Shu Ma:** investigation (equal), validation (equal). **Lingxiu Liang:** data curation (equal), software (equal). **Wangchuan Zhang:** data curation (equal). **Xia Lu:** conceptualization (equal), investigation (equal).

## Conflicts of Interest

The authors declare no conflicts of interest.

## Supporting information


**Table S1:** Primer information and protocols of PCR for *Mongoloniscus sinensis* .
**Table S2:** Nucleotide polymorphism in each population group of *Mongoloniscus sinensis* .
**Table S3:** Historical gene flow as estimated by Migrate‐N. The groups East, West, and Midland correspond to clusters identified in the genetic structure analysis.
**Table S4:** The relative contributions of the environmental variables to the Maxent model for determining the distribution of *M. sinensis* .

## Data Availability

The datasets are available at Figshare https://doi.org/10.6084/m9.figshare.28824062.

## References

[ece372318-bib-0001] Allio, R. , S. Donega , N. Galtier , and B. Nabholz . 2017. “Large Variation in the Ratio of Mitochondrial to Nuclear Mutation Rate Across Animals: Implications for Genetic Diversity and the Use of Mitochondrial DNA as a Molecular Marker.” Molecular Biology and Evolution 34, no. 11: 2762–2772. 10.1093/molbev/msx197. 28981721

[ece372318-bib-0002] Beerli, P. , and J. Felsenstein . 2001. “Maximum Likelihood Estimation of a Migration Matrix and Effective Population Sizes in n Subpopulations by Using a Coalescent Approach.” Proceedings of the National Academy of Sciences of the United States of America 98: 4563–4568. 10.1073/pnas.081068098. 11287657 PMC31874

[ece372318-bib-0003] Bensasson, D. , D. X. Zhang , D. L. Hartl , and G. M. Hewitt . 2001. “Mitochondrial Pseudogenes: Evolution's Misplaced Witnesses.” Trends in Ecology & Evolution 16, no. 6: 314–321. 10.1016/S0169-5347(01)02151-6. 11369110

[ece372318-bib-0004] Bouckaert, R. , J. Heled , D. Kühnert , et al. 2014. “BEAST 2: a Software Platform for Bayesian Evolutionary Analysis.” PLoS Computational Biology 10: e1003537. 10.1371/journal.pcbi.1003537. 24722319 PMC3985171

[ece372318-bib-0005] Brandão, C. R. F. , and M. A. R. C. de Mello . 2018. “Molecular Phylogeny and Divergence Times of Oniscidea (Isopoda) Based on Mitochondrial COI Gene: Implications for Historical Biogeography.” Journal of Zoological Systematics and Evolutionary Research 56: 12.

[ece372318-bib-0007] Chen, G. X. 2000. “Terrestrial Isopoda Fauna of Typical Zones in China.” Acta Zoologica Sinica 46: 255–264.

[ece372318-bib-0008] Chen, T. Y. , and A. R. Lou . 2019. “Phylogeography and Paleodistribution Models of a Widespread Birch ( *Betula platyphylla* Suk.) Across East Asia: Multiple Refugia, Multidirectional Expansion, and Heterogeneous Genetic Pattern.” Ecology and Evolution 9: 7792–7807. 31346441 10.1002/ece3.5365 PMC6635942

[ece372318-bib-0009] Cheng, L. , T. R. Connor , J. Sirén , D. M. Aanensen , and J. Corander . 2013. “Hierarchical and Spatially Explicit Clustering of DNA Sequences With BAPS Software.” Molecular Biology and Evolution 30: 1224–1228. 10.1093/molbev/mst028. 23408797 PMC3670731

[ece372318-bib-0010] Chou, Y. , P. Thomas , X. Ge , B. LePage , and C. Wang . 2011. “Refugia and Phylogeography of Taiwania in East Asia.” Journal of Biogeography 38: 1992–2005. 10.1111/j.1365-2699.2011.02537.x.

[ece372318-bib-0011] Clark, P. U. , and A. C. Mix . 2002. “Ice Sheets and Sea Level of the Last Glacial Maximum.” Quaternary Science Reviews 21: 1–7. 10.1016/S0277-3791(01)00118-4.

[ece372318-bib-0012] Corbalán, V. , M. F. Tognelli , J. A. Scolaro , and S. A. Roig‐Juñent . 2011. “Lizards as Conservation Targets in Argentinean Patagonia.” Journal for Nature Conservation 19: 60–67. 10.1016/j.jnc.2010.05.004.

[ece372318-bib-0013] Curnow, R. N. , and S. D. Wright . 1978. Evolution and the Genetics of Populations, Volume 4: Variability Within and Among Natural Populations. University of Chicago Press.

[ece372318-bib-0014] Cutter, A. D. 2013. “Integrating Phylogenetics, Phylogeography and Population Genetics Through Genomes and Evolutionary Theory.” Molecular Phylogenetics and Evolution 69: 1172–1185. 10.1016/j.ympev.2013.06.006. 23800835

[ece372318-bib-0015] Dallaire, X. , E. Normandeau , T. Brazier , et al. 2025. “Leveraging Whole Genomes, Mitochondrial DNA and Haploblocks to Decipher Complex Demographic Histories: An Example From a Broadly Admixed Arctic Fish.” Molecular Ecology 34, no. 10: e17772. 40289656 10.1111/mec.17772 PMC12051761

[ece372318-bib-0016] de Oliveira, F. F. R. , M. Gehara , M. Solé , et al. 2021. “Quaternary Climatic Fluctuations Influence the Demographic History of Two Species of Sky‐Island Endemic Amphibians in the Neotropics.” Molecular Phylogenetics and Evolution 160: 107113. 10.1016/j.ympev.2021.107113. 33610648

[ece372318-bib-0017] Dimitriou, A. C. , A. Antoniou , I. Alexiou , N. Poulakakis , A. Parmakelis , and S. Sfenthourakis . 2022. “Diversification Within an Oceanic Mediterranean Island: Insights From a Terrestrial Isopod.” Molecular Phylogenetics and Evolution 175: 107585. 10.1016/j.ympev.2022.107585. 35810970

[ece372318-bib-0018] Drummond, A. J. , and A. Rambaut . 2007. “BEAST: Bayesian Evolutionary Analysis by Sampling Trees.” BMC Evolutionary Biology 7: 214. 10.1186/1471-2148-7-214. 17996036 PMC2247476

[ece372318-bib-0019] Excoffier, L. , and H. E. Lischer . 2010. “Arlequin Suite ver 3.5: a New Series of Programs to Perform Population Genetics Analyses Under Linux and Windows.” Molecular Ecology Resources 10: 564–567. 10.1111/j.1755-0998.2010.02847.x. 21565059

[ece372318-bib-0020] Fang, F. , Y. Ji , Q. Zhao , et al. 2015. “Phylogeography of the Chinese Endemic Freshwater Crab *Sinopotamon Acutum* (Brachyura, Potamidae).” Zoologica Scripta 44: 653–666. 10.1111/zsc.12131.

[ece372318-bib-0021] Fu, T. , L. Han , H. Gao , H. Liang , L. Xiao , and J. Liu . 2018. “Pedodiversity and Its Controlling Factors in Mountain Regions ‐ A Case Study of Taihang Mountain, China.” Geoderma 310: 230–237. 10.1016/j.geoderma.2017.09.027.

[ece372318-bib-0022] Fu, Y. X. 1997. “Statistical Tests of Neutrality of Mutations Against Population Growth, Hitchhiking and Background Selection.” Genetics 147: 915–925. 10.1093/genetics/147.2.915. 9335623 PMC1208208

[ece372318-bib-0023] Gao, E. , J. He , Z. Wang , Y. Xu , X. Tang , and H. Jiang . 2017. “China's Zoogeographical Regionalization Based on Terrestrial Vertebrates.” Biodiversity Science 25: 9. 10.17520/biods.2017135.

[ece372318-bib-0024] Geng, S. , P. Shi , N. Zong , and W. Zhu . 2018. “Using Soil Survey Database to Assess Soil Quality in the Heterogeneous Taihang Mountains, North China.” Sustainability 10: 3443. 10.3390/su10103443.

[ece372318-bib-0025] Haag‐Liautard, C. , N. Coffey , D. Houle , M. Lynch , B. Charlesworth , and P. D. Keightley . 2008. “Direct Estimation of the Mitochondrial DNA Mutation Rate in *Drosophila melanogaster* .” PLoS Biology 6: e204. 10.1371/journal.pbio.0060204. 18715119 PMC2517619

[ece372318-bib-0026] Hawlitschek, O. , L. Hendrich , M. Espeland , E. F. Toussaint , M. J. Genner , and M. Balke . 2012. “Pleistocene Climate Change Promoted Rapid Diversification of Aquatic Invertebrates in Southeast Australia.” BMC Evolutionary Biology 12: 142. 10.1186/1471-2148-12-142. 22873814 PMC3503846

[ece372318-bib-0027] He, J. , H. Kreft , E. Gao , Z. Wang , and H. Jiang . 2017. “Patterns and Drivers of Zoogeographical Regions of Terrestrial Vertebrates in China.” Journal of Biogeography 44: 1172–1184. 10.1111/jbi.12892.

[ece372318-bib-0028] Hewitt, G. 2000. “The Genetic Legacy of the Quaternary Ice Ages.” Nature 405: 907–913. 10.1038/35016000. 10879524

[ece372318-bib-0029] Hewitt, G. M. 2004. “Genetic Consequences of Climatic Oscillations in the Quaternary.” Philosophical Transactions of the Royal Society of London. Series B, Biological Sciences 359: 183–195. 10.1098/rstb.2003.1388. 15101575 PMC1693318

[ece372318-bib-0030] Hoorn, C. , F. P. Wesselingh , H. ter Steege , et al. 2010. “Amazonia Through Time: Andean Uplift, Climate Change, Landscape Evolution, and Biodiversity.” Science 330: 927–931. 10.1126/science.1194585. 21071659

[ece372318-bib-0032] Hou, Z. , J. Li , and S. Li . 2014. “Diversification of Low Dispersal Crustaceans Through Mountain Uplift: a Case Study of *Gammarus* (Amphipoda: Gammaridae) With Descriptions of Four Novel Species.” Zoological Journal of the Linnean Society 170: 591–633. 10.1111/zoj.12119.

[ece372318-bib-0033] Huson, D. H. , and D. Bryant . 2006. “Application of Phylogenetic Networks in Evolutionary Studies.” Molecular Biology and Evolution 23: 254–267. 10.1093/molbev/msj030. 16221896

[ece372318-bib-0034] Karagkouni, M. , S. Sfenthourakis , A. Feldman , and S. Meiri . 2016. “Biogeography of Body Size in Terrestrial Isopods (Crustacea: Oniscidea).” Journal of Zoological Systematics and Evolutionary Research 54: 182–188. 10.1111/jzs.12125.

[ece372318-bib-0035] Katoh, K. , and D. M. Standley . 2013. “MAFFT Multiple Sequence Alignment Software Version 7: Improvements in Performance and Usability.” Molecular Biology and Evolution 30: 772–780. 23329690 10.1093/molbev/mst010 PMC3603318

[ece372318-bib-0036] Kearse, M. , R. Moir , A. Wilson , et al. 2012. “Geneious Basic: an Integrated and Extendable Desktop Software Platform for the Organization and Analysis of Sequence Data.” Bioinformatics 28: 1647–1649. 10.1093/bioinformatics/bts199. 22543367 PMC3371832

[ece372318-bib-0037] Kreft, H. , and W. Jetz . 2013. “Comment on ‘An Update of Wallace's Zoogeographic Regions of the World’.” Science 341: 343. 10.1126/science.1237471. 23888023

[ece372318-bib-0038] Lei, Q. , D. K. Liu , S. Li , and W. Ji . 2019. “Geomorphological Characteristics and Cause of Taihang Mountains.” Technology Innovation and Application 9: 78–79.

[ece372318-bib-0039] Leigh, J. W. , and D. Bryant . 2015. “Popart: Full‐Feature Software for Haplotype Network Construction.” Methods in Ecology and Evolution 6: 1110–1116. 10.1111/2041-210X.12410.

[ece372318-bib-0040] Li, J. X. , L. P. Yue , C. Y. Liu , X. Y. Wang , and G. F. Li . 2013. “The Tectonic‐Sedimentary Evolution of the Lüliang Mountains Since the Miocene (In Chinese).” Journal of Stratigraphy 37: 93–100.

[ece372318-bib-0041] Librado, P. , and J. Rozas . 2009. “DnaSP v5: a Software for Comprehensive Analysis of DNA Polymorphism Data.” Bioinformatics 25: 1451–1452. 10.1093/bioinformatics/btp187. 19346325

[ece372318-bib-0042] Lin, N. , J. B. Landis , Y. Sun , et al. 2021. “Demographic History and Local Adaptation of *Myripnois Dioica* (Asteraceae) Provide Insight on Plant Evolution in Northern China Flora.” Ecology and Evolution 11: 8000–8013. 10.1002/ece3.7628. 34188867 PMC8216978

[ece372318-bib-0043] Liu, W. , Y. Zhao , D. Qi , J. You , Y. Zhou , and Z. Song . 2018. “The Tanggula Mountains Enhance Population Divergence in Carex Moorcroftii: a Dominant Sedge on the Qinghai‐Tibetan Plateau.” Scientific Reports 8, no. 1: 2741. 29426823 10.1038/s41598-018-21129-y PMC5807306

[ece372318-bib-0044] Ma, R. , L. Zhang , Y. Xu , C. Wei , and H. He . 2024. “The Influence of Climate Oscillations and Geological Events on Population Differentiation of *Camponotus Japonicus* in the Chinese Mainland.” Ecology and Evolution 14: e11077. 10.1002/ece3.11077. 38390001 PMC10883248

[ece372318-bib-0045] Muraro, V. , M. S. Mazzochi , A. M. C. R. Fregonezi , and L. Bugoni . 2024. “Role of Environmental Factors in the Genetic Structure of a Highly Mobile Seabird.” Journal of Biogeography 51: 1933–1943. 10.1111/jbi.14862.

[ece372318-bib-0046] Pakendorf, B. , and M. Stoneking . 2005. “Mitochondrial DNA and Human Evolution.” Annual Review of Genomics and Human Genetics 6: 165–183. 10.1146/annurev.genom.6.080604.162249. 16124858

[ece372318-bib-0047] Paoletti, M. G. , and M. Hassall . 1999. “Woodlice (Isopoda: Oniscidea): Their Potential for Assessing Sustainability and Use as Bioindicators.” Agriculture, Ecosystems & Environment 74: 157–165.

[ece372318-bib-0048] Petit, R. , I. Aguinagalde , J. L. de Beaulieu , et al. 2003. “Glacial Refugia: Hotspots but Not Melting Pots of Genetic Diversity.” Science 300: 1563–1565. 10.1126/science.1083264. 12791991

[ece372318-bib-0049] Phillips, S. J. , R. P. Anderson , and R. E. Schapire . 2006. “Maximum Entropy Modeling of Species Geographic Distributions.” Ecological Modelling 190: 231–259. 10.1016/j.ecolmodel.2005.03.026.

[ece372318-bib-0050] Podsiadlowski, L. , and T. Bartolomaeus . 2005. “Organization of the Mitochondrial Genome of Mantis Shrimp *Pseudosquilla ciliata* (Crustacea: Stomatopoda).” Marine Biotechnology 7: 618–624. 10.1007/s10126-005-0017-8. 16088353

[ece372318-bib-0051] Promnun, P. , N. Tandavanitj , C. Kongrit , et al. 2021. “Phylogeography and Ecological Niche Modeling Reveal Evolutionary History of *Leiolepis Ocellata* (Squamata, Leiolepidae).” Ecology and Evolution 11: 2221–2233. 10.1002/ece3.7186. 33717450 PMC7920770

[ece372318-bib-0052] Qu, Y. , F. Lei , R. Zhang , and X. Lu . 2010. “Comparative Phylogeography of Five Avian Species: Implications for Pleistocene Evolutionary History in the Qinghai‐Tibetan Plateau.” Molecular Ecology 19: 338–351. 10.1111/j.1365-294X.2009.04445.x. 20002586

[ece372318-bib-0053] Rato, C. , L. B. Sreelatha , F. Gómez‐Ramírez , and M. A. Carretero . 2024. “A Pleistocene Biogeography in Miniature: The Small‐Scale Evolutionary History of *Podarcis lusitanicus* (Squamata, Lacertidae).” Journal of Biogeography 0: 1–13. 10.1111/jbi.15026.

[ece372318-bib-0054] Remi, A. , D. Stefano , G. Nicolas , and N. Benoit . 2017. “Large Variation in the Ratio of Mitochondrial to Nuclear Mutation Rate Across Animals: Implications for Genetic Diversity and the Use of Mitochondrial DNA as a Molecular Marker.” Molecular Biology and Evolution 34, no. 11: 2762–2772. 28981721 10.1093/molbev/msx197

[ece372318-bib-0055] Ren, L. , Y. Su , C. Ba , and X. Liu . 2005. “PCR Primer Design Techniques.” Modern Journal of Animal Husbandry and Veterinary Medicine 6: 1.

[ece372318-bib-0056] Rogers, A. R. , and H. Harpending . 1992. “Population Growth Makes Waves in the Distribution of Pairwise Genetic Differences.” Molecular Biology and Evolution 9: 552–569. 10.1093/oxfordjournals.molbev.a040727. 1316531

[ece372318-bib-0057] Schmidt, C. 2008. “Phylogeny of the Terrestrial Isopoda (Oniscidea): A Review.” Arthropod Systematics & Phylogeny 66: 191–226.

[ece372318-bib-0058] Sfenthourakis, S. , and S. Taiti . 2015. “Patterns of Taxonomic Diversity Among Terrestrial Isopods.” ZooKeys 515: 13–25. 10.3897/zookeys.515.9332. PMC4525032 26261437

[ece372318-bib-0059] Stewart, J. R. , A. M. Lister , I. Barnes , and L. Dalén . 2010. “Refugia Revisited: Individualistic Responses of Species in Space and Time.” Proceedings of the Royal Society B: Biological Sciences 277: 661–671. 10.1098/rspb.2009.1272 PMC2842738 19864280

[ece372318-bib-0061] Taiti, S. 2004. Crustacea: Isopoda: Oniscidea (Woodlice), 547–550. Encyclopedia of Caves and Karst Science. Routledge.

[ece372318-bib-0062] Tajima, F. 1989. “Statistical Method for Testing the Neutral Mutation Hypothesis by DNA Polymorphism.” Genetics 123: 585–595. 10.1093/genetics/123.3.585. 2513255 PMC1203831

[ece372318-bib-0085] Talavera, G. , and J. Castresana . 2007. “Improvement of Phylogenies After Removing Divergent and Ambiguously Aligned Blocks from Protein Sequence Alignments.” Systematic Biology 56, no. 4: 564–577. 10.1080/10635150701472164. 17654362

[ece372318-bib-0063] Tang, X.‐T. , M.‐X. Lu , and Y.‐Z. Du . 2022. “Molecular Phylogeography and Evolutionary History of the Pink Rice Borer (Lepidoptera: Noctuidae): Implications for Refugia Identification and Pest Management.” Systematic Entomology 47: 371–383. 10.1111/syen.12535.

[ece372318-bib-0064] Veríssimo, J. , M. Znari , H. Stuckas , et al. 2016. “Pleistocene Diversification in Morocco and Recent Demographic Expansion in the Mediterranean Pond Turtle *Mauremys Leprosa* .” Biological Journal of the Linnean Society 119, no. 4: 943–959. 10.1111/bij.12849.

[ece372318-bib-0065] Vieira, P. E. , A. Desiderato , D. M. Holdich , et al. 2019. “Deep Segregation in the Open Ocean: Macaronesia as an Evolutionary Hotspot for Low Dispersal Marine Invertebrates.” Molecular Ecology 28, no. 7: 1784–1800. 30768810 10.1111/mec.15052

[ece372318-bib-0066] Webb, T. , and P. J. Bartlein . 1992. “Global Changes During the Last 3 Million Years: Climatic Controls and Biotic Responses.” Annual Review of Ecology, Evolution, and Systematics 23: 141–173.

[ece372318-bib-0067] Wiens, J. J. , and M. J. Donoghue . 2004. “Historical Biogeography, Ecology and Species Richness.” Trends in Ecology & Evolution 19: 639–644. 10.1016/j.tree.2004.09.011. 16701326

[ece372318-bib-0068] Wu, C. , X. Zhang , and Y. Ma . 1999. “The Taihang and Yan Mountains Rosemainly in Quarteranary.” North China Earthquake Sciences 17: 7.

[ece372318-bib-0086] Xiong, Y. , Y. L. Xiong , X. J. Jia , et al. 2024. “Divergence in *Elymus sibiricus* is Related to Geography and Climate Oscillation: A New Look from Pan‐Chloroplast Genome Data.” Journal of Systematics and Evolution 62: 794–808. 10.1111/jse.13020.

[ece372318-bib-0069] Xu, H. , L. Huang , T. Chen , et al. 2024. “Phylogeography and Population Structure of *Lagocephalus spadiceus* (Richardson, 1845) (Tetraodontiformes, Tetraodontidae) in the South China Sea.” Ecology and Evolution 14, no. 4: e11320. 38681184 10.1002/ece3.11320 PMC11045559

[ece372318-bib-0070] Xu, X. , M. Kuntner , F. Liu , J. Chen , and D. Li . 2018. “Formation of Rivers and Mountains Drives Diversification of Primitively Segmented Spiders in Continental East Asia.” Journal of Biogeography 45: 2080–2091. 10.1111/jbi.13403.

[ece372318-bib-0071] Xu, X. X. , F. Y. Cheng , L. P. Peng , et al. 2019. “Late Pleistocene Speciation of Three Closely Related Tree Peonies Endemic to the Qinling‐Daba Mountains, a Major Glacial Refugium in Central China.” Ecology and Evolution 9, no. 13: 7528–7548. 10.1002/ece3.5284. 31346420 PMC6635923

[ece372318-bib-0072] Ye, H. , Y. Wang , H. Liu , et al. 2024. “The Phylogeography of Deciduous Tree *Ulmus Macrocarpa* (Ulmaceae) in Northern China.” Plants 13: 1334. 10.3390/plants13101334. 38794406 PMC11125379

[ece372318-bib-0073] Ye, Z. , J. Yuan , Y. Zhen , et al. 2020. “Local Environmental Selection and Lineage Admixture Act as Significant Mechanisms in the Adaptation of the Widespread East Asian Pond Skater *Gerris Latiabdominis* to Heterogeneous Landscapes.” Journal of Biogeography 47: 1154–1165. 10.1111/jbi.13774.

[ece372318-bib-0074] Yu, D. , M. Chen , Q. Tang , X. Li , and H. Liu . 2014. “Geological Events and Pliocene Climate Fluctuations Explain the Phylogeographical Pattern of the Cold Water Fish *Rhynchocypris Oxycephalus* (Cypriniformes: Cyprinidae) in China.” BMC Evolutionary Biology 14: 225. 10.1186/s12862-014-0225-9. 25344323 PMC4219125

[ece372318-bib-0075] Zhang, D. , F. Gao , I. Jakovlić , et al. 2019. “PhyloSuite: An Integrated and Scalable Desktop Platform for Streamlined Molecular Sequence Data Management and Evolutionary Phylogenetics Studies.” Molecular Ecology Resources 20: 348–355. 10.1111/1755-0998.13096. 31599058

[ece372318-bib-0076] Zhang, Y. , Y. Ma , N. Yang , W. Shi , and S. Dong . 2003. “Cenozoic Extensional Stress Evolution in North China.” Journal of Geodynamics 36, no. 5: 591–613.

[ece372318-bib-0077] Zhao, H. , Q. R. Wang , W. Fan , and G. H. Song . 2017. “The Relationship Between Secondary Forest and Environmental Factors in the Southern Taihang Mountains.” Scientific Reports 7: 16431. 10.1038/s41598-017-16647-0. 29180781 PMC5703897

[ece372318-bib-0078] Zhao, J. F. , C. Y. Liu , X. M. Wang , Y. P. Ma , and L. Huang . 2009. “Uplifting and Evolution Characteristics of the Lüliang Mountains and Its Adjacent Area During Meso‐Cenozoic (In Chinese).” Geological Review 55: 673–682.

[ece372318-bib-0079] Zhao, Q. , H. X. Liu , L. G. Luo , and X. Ji . 2011. “Comparative Population Genetics and Phylogeography of Two Lacertid Lizards (*Eremias Argus* and *E. Brenchleyi*) From China.” Molecular Phylogenetics and Evolution 58: 478–491. 10.1016/j.ympev.2010.12.017. 21215808

[ece372318-bib-0080] Zhao, Q. , E. Shi , Y. Li , R. Eberl , and J. An . 2016. “Population Genetic Structure and Demographic History of the Chinese Endemic *Mongoloniscus sinensis* (Dollfus, 1901) (Isopoda: Oniscidea).” Zoological Systematics 41: 352–365. 10.11865/Zs.201641.

[ece372318-bib-0081] Zhao, T. , M. Wang , M. Li , and J. An . 2019. “Toxicity of Heavy Metals to *Mongoloniscus sinensis* (Dollfus, 1901) (Crustacea: Isopoda: Oniscidea).” Bulletin of Environmental Contamination and Toxicology 102: 25–31. 10.1007/s00128-018-2480-8. 30382304

[ece372318-bib-0082] Zhao, Y. , Z. Qi , W. Ma , et al. 2013. “Comparative phylogeography of the *Smilax hispida* group (Smilacaceae) in eastern Asia and North America – implications for allopatric speciation, causes of diversity disparity, and origins of temperate elements in Mexico.” Molecular Phylogenetics and Evolution 68: 300–311. 10.1016/j.ympev.2013.03.025. 23578597

[ece372318-bib-0083] Zhu, L. , F. Zhang , and M. Zhu . 2008. “Spider Community Structure in Typical Habitats of Xiaowutai Mountain, China.” Journal of Hebei Univiersity (Natural Science Edition) 28: 4.

[ece372318-bib-0084] Zong, Y. , P. Sun , X. Y. Yue , Q. F. Niu , and Y. W. Teng . 2017. “Variation in Microsatellite Loci Reveals a Natural Boundary of Genetic Differentiation Among *Pyrus Betulaefolia* Populations in Northern China.” Journal of the American Society for Horticultural Science 142, no. 5: 319–329.

