# Supplementary material for: Population Genetics of an Endemic Species Mongoloniscus sinensis (Isopoda: Oniscidea) and Diversification Dynamics Across Northern China
Source: Ecol Evol. 2025 Oct 11;15(10):e72318. doi: 10.1002/ece3.72318 (PMC12514759; doi:10.1002/ece3.72318)
Supplement: Supplementary file 1 — Table S1: Primer information and protocols of PCR for Mongoloniscus sinensis . Table S2: Nucleotide polymorphism in each population group of Mongoloniscus sinensis . Table S3: Historical gene flow as estimated by Migrate‐N. The groups East, West, and Midland correspond to clusters identified in the genetic structure analysis. Table S4: The relative contributions of the environmental variables to the Maxent model for determining the distribution of M. sinensis . [file ECE3-15-e72318-s001.docx]

**Supporting information**

**Population genetics of an endemic species *Mongoloniscus sinensis* (Isopoda: Oniscidea) and diversification dynamics across northern China**

**Table S1** Primer information and protocols of PCR for *Mongoloniscus sinensis*

| **Gene** | **Primer (5’-3’)** | **PCR protocol** |
| --- | --- | --- |
| COI | F：GGTCAACAAATCATAAAGATATTGG | 94℃,3.5min;33×(94℃,30s;53℃,30s;72℃,1min);72℃,5min |
|  | R:TAAACTTCAGGGTGACCAAAAAATCA |  |
| 12S | F:CAGCAKYCGCGGTTAKAC | 94℃,3.5min;33×(94℃,30s;53℃,30s;72℃,1min);72℃,5min |
|  | R:ACACCTACTWTGTTACGACTTATCTC |  |
| ND5 | F:TTTATCTTTTGGGTTCGCTA | 94℃,3.5min;31×(94℃,30s;48℃,30s;72℃,1min);72℃,5min |
|  | R:TAAAATTAAATCCTTGCCCTC |  |

**Table S2** Nucleotide polymorphism in each population group of *Mongoloniscus sinensis*.

| Region | Sample size | NHap | Hd | Pi | k | Eta | S |
| --- | --- | --- | --- | --- | --- | --- | --- |
| East | 106 | 33 | 0.823 | 0.0029 | 4.102 | 59 | 57 |
| West | 133 | 66 | 0.931 | 0.0056 | 7.866 | 115 | 109 |
| Midland | 66 | 51 | 0.982 | 0.0347 | 48.844 | 285 | 256 |
| All | 305 | 148 | 0.9637 | 0.0298 | 41.997 | 329 | 294 |

NHap-number of haplotypes, Hd-haplotype diversity, Pi-nucleotide diversity, k-average number of nucleotide differences, Eta-total number of mutations, S-number of segregating sites.

**Table S3** Historical gene flow as estimated by Migrate-N. The groups East, West and Midland correspond to clusters identified in the genetic structure analysis.

|  | *θ* | *NeM* | | |
| --- | --- | --- | --- | --- |
|  |  | East→ | West→ | Midland→ |
| East | 2.5 |  | - | 13.0 |
| West | 2.6 | - |  | 35.7 |
| Midland | 4.8 | 257.7 | 68.3 |  |

Note: The mode of the posterior distribution is shown in the table; *θ*, 4× effective population size × mutation rate per site per generation; *NeM*, effective number of migrants per generation; →, source populations.

**Table S4** The relative contributions of the environmental variables to the Maxent model for determining the distributon of *M. sinensis*.

| Variable | Percent contribution | Permutation importance |
| --- | --- | --- |
| Bio11 | 44.5 | 42.8 |
| Bio19 | 20 | 2.7 |
| Elevation | 13.5 | 26.1 |
| Bio04 | 4.7 | 0.4 |
| Bio12 | 3.7 | 4.2 |
| Bio13 | 3.2 | 3.4 |
| Bio05 | 1.7 | 0.7 |
| Bio14 | 1.5 | 6.1 |
| Bio02 | 1.2 | 3.8 |
| Bio08 | 1.2 | 1.5 |
| Bio15 | 0.9 | 0.6 |
| Bio07 | 0.9 | 1.6 |
| Bio09 | 0.8 | 0 |
| Bio17 | 0.8 | 1.4 |
| Bio01 | 0.6 | 2.5 |
| Bio18 | 0.4 | 0.5 |
| Bio10 | 0.4 | 0.3 |
| Bio16 | 0 | 1 |
| Bio06 | 0 | 0.3 |
| Bio03 | 0 | 0 |
